# Lactococci and lactobacilli as mucosal delivery vectors for therapeutic proteins and DNA vaccines

**DOI:** 10.1186/1475-2859-10-S1-S4

**Published:** 2011-08-30

**Authors:** Luis G Bermúdez-Humarán, Pascale Kharrat, Jean-Marc Chatel, Philippe Langella

**Affiliations:** 1 INRA, UMR1319 Micalis, Commensal and Probiotics-Host Interactions Laboratory, Domaine de Vilvert, 78352 Jouy-en-Josas Cedex, France

## Abstract

Food-grade Lactic Acid Bacteria (LAB) have been safely consumed for centuries by humans in fermented foods. Thus, they are good candidates to develop novel oral vectors, constituting attractive alternatives to attenuated pathogens, for mucosal delivery strategies. Herein, this review summarizes our research, up until now, on the use of LAB as mucosal delivery vectors for therapeutic proteins and DNA vaccines. Most of our work has been based on the model LAB *Lactococcus lactis*, for which we have developed efficient genetic tools, including expression signals and host strains, for the heterologous expression of therapeutic proteins such as antigens, cytokines and enzymes. Resulting recombinant lactococci strains have been tested successfully for their prophylactic and therapeutic effects in different animal models: i) against human papillomavirus type 16 (HPV-16)-induced tumors in mice, ii) to partially prevent a bovine β-lactoglobulin (BLG)-allergic reaction in mice and iii) to regulate body weight and food consumption in obese mice. Strikingly, all of these tools have been successfully transposed to the *Lactobacillus* genus, in recent years, within our laboratory. Notably, anti-oxidative *Lactobacillus casei* strains were constructed and tested in two chemically-induced colitis models. In parallel, we also developed a strategy based on the use of *L. lactis* to deliver DNA at the mucosal level, and were able to show that *L. lactis* is able to modulate the host response through DNA delivery. Today, we consider that all of our consistent data, together with those obtained by other groups, demonstrate and reinforce the interest of using LAB, particularly lactococci and lactobacilli strains, to develop novel therapeutic protein mucosal delivery vectors which should be tested now in human clinical trials.

## Introduction

	The administration of therapeutic molecules via mucosal routes offers several important advantages over systemic delivery such as reduction of secondary effects, easy administration and the possibility to modulate both systemic and mucosal immune responses [1]. Moreover, direct delivery of the appropriate medical molecules to exert their effects at mucosal surfaces is a very efficient prophylactic and therapeutic strategy. Mucosal surfaces are the primary interaction sites between an organism and its environment and they thus represent the major portal of entry for pathogens. In the last fifteen years, there have been several reports of successful immunisation with a variety of mucosal vector vaccines [2]. They can induce efficient systemic immune responses with less collateral side effects than systemic vaccines [1]. Additionally, mucosal immunisation is more easily performed, without the need for needles and syringes, thereby eliminating the requirement for trained personnel (important feature for mass vaccination programs) [3]. Nevertheless, a major disadvantage is that a large amount of protein needs to be administered, due to the fact that the majority of protein will degrade, with very small quantities surviving degradation at mucosal surfaces such as the gastro intestinal tract [1]. Therefore, the development of new vectors, able to efficiently deliver molecules to target tissues represents a technological challenge.

Today, sufficient data is available supporting the fact that lactic acid bacteria (LAB), notably lactococci and lactobacilli, are excellent candidates as delivery vectors of therapeutic proteins, in the development of novel preventive and therapeutic strategies for humans. LAB are non-pathogenic Gram-positive bacteria and they have an extraordinary safety profile, since they have been widely consumed for centuries by humans in fermented foods. Therefore, they constitute an attractive alternative to attenuated pathogens, which are the most popular live vectors being used currently. Attenuated pathogenic bacteria, such as derivatives of *Mycobacterium*, *Salmonella*, and *Bordetella* spp. are particularly well adapted to interact with mucosal surfaces as most of them they normally use to initiate the infection process. Unfortunately, these bacteria can recover their pathogenic potential and are therefore not entirely safe for use in humans, especially in children and immunosuppressed patients. Several detailed reviews of vector delivery strategies, based on LAB, have been published within the last five years, of which three are particularly exhaustive and convincing [4-6]. Herein, we will be summarizing our current research and advances on the use of lactococci and lactobacilli as live delivery vectors of proteins with health interest. We will also be describing the use of LAB as DNA-vaccine-delivery vehicles to deliver DNA directly to antigen-presenting cells of the immune system.

### 1. Lactic acid bacteria as mucosal delivery vectors of antigens and cytokines

	The immunogenicity of soluble proteins administered orally or intranasally is generally low and can be significantly enhanced by either coupling the protein to a bacterial carrier or by genetic engineering of bacteria resulting in the production of the desired antigen. As previously mentioned, food-grade or commensal Gram-positive bacteria constitute an attractive alternative to attenuated pathogenic bacteria [6]. In particular, the model LAB *Lactococcus lactis* and certain species of lactobacilli possess a number of properties, making them attractive candidates for the development of mucosal vaccines [7]. Moreover, many antigens and/or cytokines have been successfully expressed in LAB, and mucosal administration of these genetically engineered LAB has been shown to elicit both systemic and mucosal immunity (see additional file 1).

	The production of a desired antigen by LAB can occur in three different cellular locations: i) intracellular, which allows the protein to escape harsh external environmental conditions (such as gastric juices in the stomach after oral administration of the recombinant strain) but requires cellular lysis for protein release and delivery, ii) extracellular, which allows the release of the protein into the external medium, resulting in direct interaction with the environment (food product or the digestive tract), and iii) cell wall-anchored, which combines the advantages of the other two locations (i.e., interaction between the cell wall-anchored protein and the environment, in addition to protection from proteolytic degradation). In this context, several studies have compared the production of different antigens in LAB, using all three locations and evaluated the subsequent immunological impact [Reviewed in Refs. [6] and [7]]. Even if the comparison between the localization is difficult due to the amount of protein depending on the localization, these studies demonstrated that the highest immune response was usually obtained with cell-wall anchored antigens exposed to the surface of LAB. Therefore, most of the recent LAB vaccination studies have selected surface exposure of the antigen of interest, rather than intra- or extracellular production.

#### *Lactococcus lactis *as live delivery vector of proteins of health interest

*L. lactis* is the most widely used LAB in the production of fermented milk products, and is considered as the model LAB because many genetic tools have been developed in particular for heterologous protein production [8]. Moreover, *L. lactis* is considered to be a good candidate for heterologous protein production because it secretes relatively few proteins and only one, Usp45, in detectable quantities [9,10]. In addition, the most commonly used laboratory *L. lactis* strain (MG1363) is plasmid-free and does not produce any extracellular proteases [11]. However, the major advantage of using *L. lactis* as a live vector for mucosal delivery of therapeutic proteins resides in its extraordinary safety profile, since this bacterium is catalogued as a non-invasive and non-pathogenic organism with a GRAS status. Finally, the capacity of *L. lactis* to produce many different proteins of health interest has been clearly demonstrated in the last two decades (see additional file 1). All of these features explain why most of the relevant studies focusing on the use of LAB as protein and DNA delivery vectors have been performed with *L. lactis*.

#### Heterologous proteins production in *L. lactis*

Currently, several inducible and constitutive expression signals are available for *L. lactis*[2][3,6]. In our studies, we mainly used the nisin inducible promoter (PnisA), which is the major element of the NICE (Nisin Induced Controlled Expression) system [12]. Nisin is a bacteriocin produced by *L. lactis*, which contains eleven adjacent chromosomal genes (*nisABTCIPRKFE*G) encoding for biosynthesis and immunity against nisin [13]. The *nisA* gene encodes for the structural nisin gene, whereas *nisRK* encode for the dual-component system responsible for the induction of other genes within the cluster. All of our genes of interest were cloned downstream of P_nisA_ and the resulting plasmid was introduced in *L. lactis* NZ9000 (MG1363 strain carrying *nisRK* genes on its chromosome). Addition of sub-inhibitor nisin concentration levels, into the culture medium, induces the expression of the gene of interest proportionally to the dose of nisin used. This system is now considered as the most efficient one for heterologous expression in *L. lactis*[14]. We have thus developed an efficient heterologous protein production-secretion system in *L. lactis* based on P_nisA_ and a small stable and well-characterized protein, *Staphylococcus aureus* nuclease (Nuc) [15].

A family of three expression vectors: pCYT, pSEC, and pCWA was developed, to allow protein targeting to be either intracellular, secreted, or cell wall anchored, respectively. We also constructed a fourth expression vector called pSEC:LEISS, which contains a synthetic propeptide (LEISSTCDA) identified as a production-secretion booster [16]. These vectors, also functional in several other LAB species including lactobacilli, streptococci, enterococci and bifidobacteria, have been successfully used to produce approximately 50 different heterologous proteins in *L. lactis* up to date (see additional file 1). More recently, we also developed a new bile salts-inducible promoter, which is currently being tested in *in vivo* experiments (data not shown). Concerning the possible host factors affecting production-secretion in *L. lactis*, we have identified *ybdD* which, once inactivated, induces an overproduction of secreted protein [Morello *et al.*, unpublished data]. In addition, the secretion machinery for *L. lactis* has also been complemented with *B. subtilis* SecDF, which induced an increase in both production and secretion rates [17]. Within our panel of *L. lactis* strains, we also have three mutants: one inactivated in the unique extracellular housekeeping protease HtrA [18], one inactivated in the major intracellular protease ClpP, and one inactivated in both HtrA and Clp [19]. These strains are essential to reach controlled and stable production of highly degraded proteins in the wild type *L. lactis* strain [20].

### 2. Use of recombinant lactococcci to induce mucosal and systemic immune responses against bacterial and viral pathogens

Currently, a number of studies support the use of recombinant *L. lactis* to induce mucosal and systemic immune responses against a desired antigen [2,7]. In 1990, the first attempt to use *L. lactis* as a mucosal vaccine was performed with killed recombinant lactococci producing a cell wall-attached form of a *Streptococcus mutans* protective antigen (PAc). Mice immunized orally with this killed *L. lactis* recombinant strain developed PAc-specific serum IgG and mucosal IgA antibodies [21]. These results demonstrated, for the first time, that *L. lactis* can efficiently present an antigen to the immune system. In 1993, Wells et al. [22] then reported, for the first time, of the use of live recombinant *L. lactis*, producing tetanus fragment C (TTFC), to protect mice via subcutaneous injection against a lethal challenge with tetanus toxin. Afterwards, the same group evaluated the effect of immunization route (oral or nasal administration) on live recombinant lactococci producing TTFC in mice [23,24]. Oral immunization in mice resulted in a lower serum IgG and mucosal IgA antibody response as compared to nasal immunisation; whereas the protective efficacy (*i.e.* challenge with tetanus toxin) was similar between both routes. Many studies have been conducted to analyze the expression of viral, bacterial or eukaryotic heterologous proteins in *L. lactis* (see additional file 1).

The immunogenicity of the resulting recombinant strains has been evaluated, in mouse models in some cases, with very promising results. Amongst them, one of the best documented projects is based on the use of recombinant *L. lactis* producing human papillomavirus type 16 (HPV-16) E7 antigen. This viral protein is considered as a major candidate antigen for vaccines against HPV-related cervical cancer, the second cause of cancer death in women. The intracellular production of E7 antigen led to its rapid degradation in the cytoplasm of *L. lactis*, even when produced in a protease-free *clpP* strain [25]. In contrast, secreted and cell wall-anchored forms are rescued from proteolysis and produced a higher level of E7 in *L. lactis*[25,26]. Antigen-specific humoral (production of E7 antibodies) and cellular (secretion of IL-2 and IFN-γ cytokines) responses were observed after intranasal administration of recombinant lactococci expressing E7 antigen at different levels and in cellular locations to mice. The responses were significantly higher in mice immunized with *L. lactis* expressing E7 as a cell wall-anchored form [27]. Subsequently, the protective effect of mucosally co-administered live *L. lactis* strains expressing cell wall-anchored E7 and a secreted form of interleukin-12 to treat HPV-16-induced tumors in a murine model was then evaluated [28]. When challenged with lethal levels of tumor cell line TC-1 expressing E7, 50% of pre-treated mice demonstrated complete prevention of TC-1-induced tumors. Therapeutic immunization with these recombinant strains, (i.e., 7 days after TC-1 injection) induced regression of palpable tumors in 35% of treated mice. These preclinical results suggest the feasibility of mucosal vaccination and/or immunotherapy against HPV-related cervical cancer using genetically engineered lactococci. Although most immunological studies have been performed with *L. lactis* producing TTFC and E7 antigen, the reports supporting the use of recombinant lactococci as mucosal vaccines continue to grow, and approximately more than 50 peer-reviewed publications have validated this potential to date (see additional file 1).

#### Use of recombinant lactococci in cow’s milk allergy model

Cow’s milk allergy (CMA) is a complex disorder and is the most common allergy in young infants, with an incidence rate of 2-6%, decreasing to 0.1-0.5 % in adulthood. CMA develops early in infancy within 12 to 24 months of birth, but 80-90% of affected children recover by acquiring tolerance to cow’s milk by the age of 5 years [29,30]. *L. lactis* has been engineered to produce β-lactoglobulin (BLG), one of the major allergens found in cow’s milk, resulting in LL-BLG. The recombinant BLG was produced predominantly in a soluble, intracellular, and mostly denatured form. Mucosal administration of LL-BLG strain induced BLG specific fecal IgA, although allergen-specific IgE, IgA, IgG1 or IgG2a were not detected in mice sera [31]. A similar immune response was reported after oral administration of recombinant *L. lactis* secreting a T-cell determinant IgE epitope of BLG [32]. Adel-Patient et al [33] then demonstrated that oral administration of recombinant *L. lactis* strains producing different amounts of recombinant BLG partially prevents mice from sensitization. Oral pre-treatment with these strains prevented a Th2-type immune response elicited by systemic sensitization, via reduction of specific IgE and the induction of allergen-specific IgG2a and fecal IgA antibodies. The intensity of the Th1 immune response induced correlates with the amount of recombinant BLG produced, since the most effective strains were those producing the highest amount of BLG [33].

Similar to oral administration, intranasal delivery of recombinant *L. lactis* strains did not induce the secretion of BLG specific antibodies, but elicited IFN-γ production in murine splenocytes after BLG re-stimulation. Intranasal pre-treatment of mice with LL-BLG reduced airway eosinophilia influx and IL-5 secretion in broncoalveolar lavage (BAL) after intranasal allergen challenge. In the same study, intranasal co-administration of recombinant LL-BLG and LL-IL12 elicited a protective Th1 immune-response, inhibiting the allergic response in mice without affecting specific BLG IgE secretion [34]. Elsewhere, we also showed that intranasal administration of LL-IL12 strain decreased allergy symptoms in an asthma model induced by ovalbumin [35]. The effects of intranasal administration of LL-BLG strain were also tested in a therapeutic protocol. In orally sensitized mice, intranasal administration of recombinant strain reduced IgG1 production but did not influence specific BLG IgE or IgG2a secretion. After intranasal challenge, a mild decrease in IL-4 and IL-5 secreted into BAL was detected [36].

#### Effects of intranasal administration of recombinant *L. lactis* strains secreting human leptin in *ob/ob* mice

Leptin is a 16 kDa protein encoded by the obese (*ob*) gene, and is an adipocyte-derived pleiotropic hormone that modulates a large number of physiological functions, including control of body weight and regulation of the immune system [37]. In humans, leptin plays a crucial role in regulation of body weight, as demonstrated by morbid obesity in patients with congenital mutations in either leptin or the leptin receptor gene [38-41]. When body fat increases, leptin inhibits food intake and stimulates energy expenditure to control body weight. Although leptin treatment induced remarkable weight-loss in patients with rare congenital leptin deficiency [42-45], it showed poor efficiency in most obese patients. Indeed, clinical trials involving subcutaneous administration of recombinant leptin to obese subjects indicated that a significant reduction of body weight was only observed if serum leptin concentrations were 20- to 30-fold higher than normal physiological levels [46]. This poor response was attributed in part to insufficient transport of leptin across the blood brain barrier in obese patients [47].

Since intranasal delivery is an efficient route for administration of drugs directly to the brain [48-50], we considered that intranasal leptin administration may be an interesting strategy to bypass the blood brain barrier in leptin resistant humans. Thus, the aim of our project was to measure the effect of intranasal administration of a recombinant *L. lactis* strain secreting a biologically active form of leptin (LL-LEP) in *ob*/*ob* mice. We first demonstrated that the secreted recombinant leptin is a fully biologically active hormone, by showing its capacity to stimulate a STAT3 reporter gene in HEK293 cells transfected with the Ob-Rb leptin receptor [51]. The immunomodulatory activity of the LL-LEP strain was then evaluated *in vivo* by co-expression with the *L. lactis* strain expressing human papillomavirus type-16 (HPV-16) E7 protein (LL- E7) [51]. In C57BL/6 mice immunized intranasally with LL-LEP and LL-E7 strains, the adaptive immune response was significantly higher than in mice immunized with LL-E7 only, demonstrating the adjuvanticity of leptin. We then analyzed the effect of daily intranasal administration of LL-LEP in *ob*/*ob* mice and thus observed that this treatment induced a significant reduction in body weight gain and food intake [51]. These results demonstrate that leptin can be produced and secreted in an active form by *L. lactis*, and that the LL-LEP strain regulated *in vivo* antigen-specific immune responses, as well as body weight and food consumption.

#### Immune response to antigens delivered by *Lactobacillus* spp

The use of genetically modified lactobacilli (i.e. *Lb. fermentum*, *Lb. acidophilus*, *Lb. casei* and *Lb. plantarum*) to produce heterologous proteins and to develop a new generation of mucosal vaccines was first proposed during the 90s decade [52,53]. By the end of the 90s and into the early 2000s, several laboratories had successfully utilized recombinant strains of *Lb. casei* and *Lb. plantarum* as vehicles for delivery of medically relevant proteins to mucosal surfaces, with both strains stimulating strong local immune responses [6,54]. Approximately 50 peer-reviewed publications have already been published confirming the advantages of the *Lactobacillus* genus to serve as live mucosal vaccines, since lactobacilli can persist longer in the digestive tract and some strains have probiotic properties (i.e. show health-promoting activities for humans and animals) [54]. Similar to *L. lactis*, several studies analyzing the expression of a variety of viral, bacterial or eukaryotic origin proteins in *Lb. plantarum* and *Lb. casei* have been conducted (see additional file 1). We have evaluated the immunogenicity of E7 antigen producing recombinant *Lb. plantarum* in mouse models with promising results [55].

#### Use of recombinant *Lb. casei* in cow’s milk allergy model

We recently developed a recombinant strain of *Lactobacillus casei* capable of producing BLG. The immunomodulatory potency of intranasal and oral administration of this recombinant lactobacilli on a subsequent sensitization of mice to BLG was investigated by Hazebrouck et al. [56], who analyzed the influence of the administration route on the immune response elicited by the recombinant BLG *Lb. casei* producing strain. Intranasal pre-administration of the BLG-producing *Lb. casei* enhanced BLG-specific IgG2a and IgG1 responses, but did not influence BLG-specific IgE production in sensitized mice. Unexpectedly, oral pre-administration led to a significant inhibition of BLG-specific IgE production, wheras IgG1 and IgG2a responses were not stimulated in sensitized mice. The production of IL-17 by BLG-reactivated splenocytes was similar between oral and intranasal route administrations. However in BLG-reactivated splenocytes from mice intranasally pretreated, a greater secretion level of Th1 cytokines (IFN-γ and IL-12) and Th2 cytokines (IL-4 and IL-5) was detected, suggesting a mixed Th1/Th2 cell response; whereas only production of Th1 cytokines, but not Th2 cytokines, was enhanced in BLG-reactivated splenocytes from mice orally pretreated. Those results indicate that the mode of administration of recombinant LAB may be critical for their immunomodulatory properties [56].

#### Anti-oxidative proteins delivery by *Lb. casei* in colitis-induced murine models

Inflammatory bowel diseases (IBD) constitute a group of disorders characterized by chronic and relapsing inflammation of the gastrointestinal tract (GIT). The two most common forms of IBD are Crohn’s disease and ulcerative colitis, which are associated with an influx of neutrophils and macrophages, resulting in the consequent production of inflammatory mediators such as proteases, cytokines and reactive oxygen species (ROS) [57]. ROS include the superoxide radical (O2°^-^), hydrogen peroxide (H_2_O_2_), and the hydroxyl radical (HO°) [58], which have all been demonstrated to be both cytotoxic and mutagenic (i.e. cause damage to cellular lipids, proteins and DNA) [59]. To detoxify ROS, cells have evolved protective mechanisms via antioxidant enzymes such as superoxide dismutases (SOD) and catalases (CAT), which degrade O2°^-^ and H_2_O_2_, respectively, thereby preveningt the formation of HO° [60]. Thus, therapeutic use of antioxidant enzymes to decrease ROS amount level is a promising strategy to prevent and/or cure IBD. Several studies have shown that LAB, such as lactobacilli, may play a preventative role in IBD [61,62]. Under this context, we originally demonstrated that *Lb. casei* BL23 strain can attenuate moderate Dextran Sodium Sulfate (DSS) induced colitis in mice [63]. However, the use of a recombinant *Lb. casei* BL23 strain producing manganese CAT (MnCAT) did not improve the protective effect of inflammation reduction [63]. On the other hand, other recent studies have successfully reported the use of either recombinant *Lb. gasseri* or *Lb. plantarum* strains to produce and deliver *in situ* biologically active manganese SOD (MnSOD) to treat colitis in an interleukin-10 (IL-10) knockout mouse model and in a 2,4,6-trinitrobenzene sulphonic acid (TNBS)-induced colitis in rats [64][65]. We then cloned MnSOD from *Lactococcus lactis* into *Lb. casei* BL23 to evaluate the potential increase in the aforementioned protective effect towards ROS by delivery of MnSOD [66]. We therefore compared the effect of intragastric administration of *Lb. casei* BL23 MnSOD alone or in combination with *Lb. casei* BL23 MnCAT in the murine model of DSS 3%-induced colitis. Based on histological scores, a significant reduction of caecal and colonic inflammation was observed with either administration of *Lb. casei* BL23 MnSOD alone or the co-administration of *Lb. casei* BL23 MnCAT and *Lb. casei* BL23 MnSOD. However, there was no additional improvement in inflammation reduction with the administration of *Lb. casei* BL23 MnCAT as compared to administering *Lb. casei* BL23 MnSOD alone. These results suggest that *Lb. casei* BL23 MnSOD may have an anti-inflammatory effect on gut inflammation. More recently, we demonstrated that both *Lb. casei* BL23 MnSOD and MnCAT were able to significantly attenuate TNBS-induced inflammation damage in mice as shown by higher survival rates, decreased animal weight loss, lower bacterial translocation to the liver and the prevention of damage to the large intestine [66].

### 3. Recombinant lactic acid bacteria as DNA delivery vehicles

The advantage of DNA vaccines relies in their ability to induce both cellular and humoral Th1 immune responses [67,68]. In contrast to bacteria-mediated delivery of protein antigens, bacteria-mediated delivery of DNA vaccines leads to the expression of post-translationally modified antigens by host cells resulting in presentation of conformationally restricted epitopes to the immune system [69]. As for protein delivery, the use of food-grade lactococci and lactobacilli as DNA delivery vehicles is a promising alternative to attenuated pathogens.

#### *L. lactis* is able to modulate the host immune response through cDNA delivery

Recombinant BLG is expressed mainly in denatured form with *E. coli* or *L. lactis*, whereas its production, in eukaryotic cells, is in the native conformation [31,70]. *L. lactis* strains have been used to deliver an expression cassette encoding BLG cDNA, under the transcriptional control of the CMV viral promoter, into the Caco-2 epithelial cell line. The expression cassette was inserted into a *L. lactis* replicating plasmid. Production and secretion of BLG was observed in Caco-2 cells after incubation with *L. lactis* carrying the expression plasmid, demonstrating that non invasive *L. lactis* can deliver fully functional plasmids into epithelial cells. Interestingly, no production of BLG was observed when Caco-2 cells were co-incubated with purified plasmid alone or mixed with *L. lactis*, suggesting that the plasmid requires to be inside the bacterium in order to achieve transfer into epithelial cells with subsequent BLG production [71]. After oral administration of *L. lactis* in mice, carrying the eukaryotic expression cassette encoding for BLG, both BLG cDNA and protein were detected in the small intestine 72 hours after the final administration. No BLG (protein/dna or both) was detected 6 days after the last oral administration. Mice developed a BLG specific Th1 primary immune response, characterized by a weak and transitory IgG2a serum response. In sensitized pre-treated mice, IgE and IL-5 concentrations decreased by 70 and 40%, respectively as compared to sensitized naive mice. Moreover, only splenocytes from pre-treated mice secreted IFN-γ after BLG specific re-activation [72]. The *in situ* production elicits a specific immune response protecting the mice from further sensitization with cow’s milk proteins. To our knowledge, this is the first evidence of functional genetic material transfer from food-grade transiting bacteria to a host.

#### Recombinant invasive lactococci as DNA delivery vehicles

As demonstrated with recombinant *E. coli*, invasion of the host cell is a limiting step to achieve efficient DNA vaccine delivery [73]. To increase lactococcal DNA vaccine delivery efficiency, *L. lactis* was rendered invasive by expression of the *inlA* gene of *Listeria monocytogenes*, encoding for the Internalin A surface protein, which mediates the invasion of non phagocytic cells by *L. monocytogenes*[74,75]. Once expressed by *L. lactis*, InlA can promote the internalization of lactoccocci into the human epithelial line Caco-2 *in vitro* and into enterocytes *in vivo* after oral administration to guinea pigs. In addition, *L. lactis* InlA+ can deliver a functional plasmid encoding for GFP, and about 1% of Caco-2 cells express GFP after co-culture with this strain [76]. Recombinant invasive *L. lactis* strains expressing the *Staphylococcus aureus* Fibronectin Binding Protein A encoding gene also showed a heightened ability to be internalized into mammalian cells as compared to the control strain. Consequently, both recombinant invasive strains were more efficient in eGFP expression plasmid delivery into Caco-2 cells, resulting in a higher number of GFP producing cells [77]. *In vivo*, *L. lactis* InlA+ was able to invade guinea pig enterocytes after oral administration [76].

## Conclusions and future challenges

We consider that all of our consistent data, together with those obtained from other groups (see additional file 1), reinforce the interest in using lactococci and lactobacilli strains to develop novel therapeutic protein mucosal delivery vectors, which should be tested now in human clinical trials. Therefore, a biocontainment strategy to prevent the dissemination in the environment of these genetically modified LAB should be developed before they can be used in humans as it is mentioned in a recent review on these strategies [78]. Following the demonstration that an IL-10-producing *L. lactis* strain (LL-IL10) could treat colitis in mouse models [79], Steidler *et al* developed the first biocontainment system for LL-IL10 strain in order to be allowed to start the first human clinical study using this recombinant strain. To address these safety concerns with the use of LL-IL10 in humans, the chromosomal thymidylate synthase (*thyA*) gene was replaced by the gene encoding for IL-10 to generate a thymidine auxotroph phenotype. In the absence of thymidine or thymine, the viability of the *thyA* LL-IL10 strain was reduced by several orders of magnitude and containment was validated *in vivo* in pigs [80]. A phase I clinical trial was then conducted with the *thyA* LL-IL10 strain in human patients suffering from CrohnÂ´s disease, demonstrating that the containment strategy was effective [81]. Following those positive results, a phase IIA trial was performed and a press release was published by the end of 2009 revealing that all three primary endpoints of the study have been met: i) safety and tolerability; ii) environmental containment and iii) assessment of biomarkers associated with the strain. With respect to the secondary endpoint, the clinical results did not reveal a statistically significant difference in mucosal healing versus placebo. In view of these results, the authors of this pioneering human clinical trial and other teams involved in this promising field should now consider the optimization of some aspects of their LAB delivery strategy. The improvement should be done at different levels such as the nature i) of the delivered molecule; ii) of the LAB species as *Lb. casei* for example seems to show some advantages compared to *L. lactis* and iii) of the expression system to increase the quantities of the delivered molecule *in situ*. Such efforts should and need to be continued because the future of prophylactic and therapeutic strategies based on recombinant lactococci and lactobacilli requires clear demonstration of their efficacy in such human clinical trials, which will lead to their better acceptance.

## Competing interests

The authors declare that they have no competing interests.

## Supplementary Material

Additional file 1 Summary of therapeutic proteins expressed by lactococci and lactobacilli and validated in animal studies. Click here for file
