# Supplementary material for: Lactococci and lactobacilli as mucosal delivery vectors for therapeutic proteins and DNA vaccines
Source: Microb Cell Fact. 2011 Aug 30;10(Suppl 1):S4. doi: 10.1186/1475-2859-10-S1-S4 (PMC3231930; doi:10.1186/1475-2859-10-S1-S4)
Supplement: Additional file 1 — Summary of therapeutic proteins expressed by lactococci and lactobacilli and validated in animal studies. [file 1475-2859-10-S1-S4-S1.docx]

**Table 1.** Summary of therapeutic proteins expressed by lactococci and lactobacilli and validated in animal studies.

| **Protein** | **Source** | **Vector** | **Potential on health** | **Reference** |
| --- | --- | --- | --- | --- |
| ***Bacterial*** |  |  |  |  |
| Pili | *Streptococcus agalactiae GBS* | *L. lactis* | Streptococcal meningitidis vaccine | [82] |
| LpA | *Borrelia burgdorferi* | *Lb. plantarum* | Lyme disease's vaccine | [83] |
| TTFC | *Clostridium tetani* | *L. lactis* | Tetanus vaccine | [22] |
|  |  | *Lb. plantarum* | Tetanus vaccine | [84] |
|  |  | *Lb. casei* | Tetanus vaccine | [85] |
| SpaA | *Erysipelothrix rhusiopathiae* | *L. lactis* | Swine erysipelas vaccine / Human erysipeloid vaccine | [86] |
| UreB | *Helicobacter pylori* | *L. lactis* | Chronic active gastritis vaccine | [87] |
|  |  | *Lb. plantarum* | Chronic active gastritis vaccine | [88] |
| Cag12 | *Helicobacter pylori* | *L. lactis* | Chronic active gastritis vaccine | [89] |
| FliC | *Salmonella enterica* serovar Enteritidis (SE) | *L. lactis* | Human foodborne illness vaccine | [90] |
| PAc | *Streptococcus mutans* | *L. lactis* | Inhibition of dental cariogenic S. mutans | [21] |
| M6 | *Streptococcus pyogenes* | *L. lactis* | Vaccine against skin infections and neurotic infections | [91] |
| PsaA | *Streptococcus pneumoniae* | *L. lactis* | Pneumococcal meningitis vaccine | [92] |
| PppA | *Streptococcus pneumoniae* | *L. lactis* | Pneumococcal meningitis vaccine | [93] |
| LLO | *Listeria monocytogenes* | *L. lactis* | Vaccine against listeriosis | [94] |
| ***Viral*** |  |  |  |  |
| NSP4 | Bovine coronavirus | *L. lactis* | Winter Dysentery vaccine in adult cows | [95] |
| SARS | Coronavirus | *Lb. casei* | Human severe acute respiratory syndrome vaccine | [96] |
| Spike glycoprotein S | Coronavirus | *Lb. casei* | Coronavirus gastroenteritis vaccine | [97] |
| EDIII | Dengue virus serotype 2 | *L. lactis* | Dengue hemorrhagic fever vaccine | [98] |
| V3 | Human immunodefficiency virus (HIV-1) | *L. lactis* | HIV vaccine | [99] |
| E7 | Human papillomavirus type-16 (HPV-16) | *L. lactis* | HPV-16 cervical cancer vaccine | [28] |
|  |  | *Lb. casei* | HPV-16 cervical cancer therapy | [100] |
|  |  | *Lb. plantarum* | HPV-16 cervical cancer therapy | [101] |
| E6 | Human papillomavirus type-16 (HPV-16) | *Lb. casei* | HPV-16 cervical cancer therapy | [102] |
| L1 | Human papillomavirus type-16 (HPV-16) | *L. lactis* | HPV-16 cervical cancer vaccine | [103] |
|  |  | *Lb. casei* | HPV-16 cervical cancer vaccine | [104] |
| HA | Avian influenza H5N1 virus | *L. lactis* | Avian influenza H5N1 vaccine | [105] |
| VP2 and VP3 | Infectious bursal disease virus (IBDV) | *L. lactis* | Infectious bursal disease vaccine | [106] |
| VP1 | chicken anemia virus (CAV) | *Lb. acidophilus* | CAV vaccine | [107] |
| VP2 | Porcine parvovirus | *Lb. casei* | Reproductive disease vaccine | [108] |
| VP7 | Rotavirus | *L. lactis* | Rotavirus gastroenteritis vaccine in young children | [109] |
| Pres/PreSa | Hepatitis B virus | *L. lactis* | Mucosal vaccine agains thepatitis B virus(HBV) | [110] |
| ***Cytokines*** |  |  |  |  |
| IL-2 | *Mus musculus* | *L. lactis* | Adjuvant for tetanus vaccine | [111] |
| IL-12 | *Mus musculus* | *L. lactis* | Adjuvant therapy for cervical cancer vaccine | [112] |
| IL-6 | *Mus musculus* | *L. lactis* | Adjuvant for tetanus vaccine | [111] |
| IL-10 | *Mus musculus* | *L. lactis* | Colitis therapy | [79] |
|  | *Homo sapiens* | *L. lactis* | Crohn's disease therapy | [80] |
| MIG/IP-10 | Mus musculus | *L. lactis* | New mucosal adjuvant | [113] |
| ***Others*** |  |  |  |  |
| Leptin | *Homo sapiens* | *L. lactis* | Control of body weight and regulation of the immune system | [114] |
| MSP-1 | *Plasmodium yoelii* | *L. lactis* | Malaria vaccine | [115] |
| MSA2 | *Plasmodium falciparum* | *L. lactis* | Malaria vaccine | [116] |
| BLG | *Bovine beta-lactoglobulin* | *L. lactis* | Allergy therapy | [31] |
|  |  | *Lb. casei* | Allergy therapy | [51] |
| Der p 5 allergen | *Dermatophagoides pteronyssinus* | *Lb. acidophilus* | Allergy therapy | [117] |
| CWP2 | *Giardia lamblia* | *L. lactis* | Giardiasis vaccine | [118] |
| D1 and D4 | *Aeromonas hydrophila* | *L. lactis* | Vaccine against Aeromonas hydrophila | [119] |
| MnSOD | *Lactococcus lactis* | *Lb. casei* | Antioxidative strategy against IBD | [120] |
| MnCAT | *Lactobacillus plantarum* | *Lb. casei* | Antioxidative strategy against IBD | [63] |
